# The role of exome data reanalysis in clarifying *STXBP3* associated inflammatory bowel disease and hearing loss

**DOI:** 10.1093/gastro/goaf053

**Published:** 2025-06-16

**Authors:** Lukáš Ryba, Tereza Lerchová, Jiří Bronský, Markéta Vlčková

**Affiliations:** Department of Biology and Medical Genetics, Second Faculty of Medicine, Charles University and Motol University Hospital, Prague, Czech Republic; Department of Paediatrics, Second Faculty of Medicine, Charles University and Motol University Hospital, Prague, Czech Republic; Department of Paediatrics, Second Faculty of Medicine, Charles University and Motol University Hospital, Prague, Czech Republic; Department of Biology and Medical Genetics, Second Faculty of Medicine, Charles University and Motol University Hospital, Prague, Czech Republic

## Introduction

Very-early onset inflammatory bowel disease (VEO-IBD) is a distinct form characterized by clinical symptoms appearing before the age of 6 years, with a high likelihood of monogenic etiology [1]. In 2021, Ouahed *et al*. [2] first identified association of pathogenic variants in the Syntaxin-binding protein 3 gene (*STXBP3*) with VEO-IBD and hearing impairment. This case is notable for the identification of a novel *de novo STXBP3* variant through exome data re-analysis, highlighting the importance of genetic testing in VEO-IBD. Additionally, it showcases our clinical experience in managing a patient with this condition, who achieved disease remission with the current treatment regimen.

## Case report

The index case is from a non-consanguineous Czech family with unremarkable family history. At birth, he presented with bilateral hearing loss, necessitating the placement of a cochlear implant at the age of 2 years. From 6 weeks of age onward, he was repeatedly admitted with recurrent hyponatremic dehydration and hypoalbuminemia due to persistent diarrhea. Based on these findings, protein-losing enteropathy was suspected. At 11 weeks of age, the patient underwent gastrocolonoscopy, which revealed mild colitis, particularly in the descending colon. Due to the persistence of the symptoms, a follow-up endoscopy was performed at 20 weeks of age and showed no resolution of the findings. Stool examination revealed positive fecal calprotectin levels. Treatment with corticosteroids and immunoglobulin substitution was initiated. Azathioprine treatment was started at 7 months of age due to persistent clinical issues. At the age of 2 years, a colonoscopy revealed severe pancolitis, with the disappearance of the vascular pattern, erosions, bleeding, and solitary pseudopolyps. Biological therapy with infliximab (Remicade^®^) was indicated due to the patient’s current clinical course, lack of response to prednisone and azathioprine, and the endoscopic findings. Infliximab therapy resulted in a partial clinical response, characterised by a reduction in stool frequency and the presence of less blood. A follow-up endoscopy conducted at 2.5 years of age revealed pancolitis, suggestive of Crohn’s disease, due to the presence of skip lesions (Paris classification [3]: A1a L2 B1 G1, Simple Endoscopic Score-Crohn’s disease [4]: 26). However, it is possible that these findings were influenced by the ongoing medical treatment. This prompted a transition to vedolizumab (Entyvio^®^). An improvement in the patient’s clinical condition was observed with this treatment. At the age of 3.5 years, a follow-up endoscopy revealed several dysplastic (grade 3) tubulovillous adenomas in the transverse colon. The boy underwent an early specialized procedure, and subsequent histological examination did not reveal persistent dysplastic changes. Given the persistence of disease activity, azathioprine was substituted with methotrexate. The combination of methotrexate and vedolizumab resulted in a mild improvement in the patient’s condition, allowing for the gradual cessation of corticosteroid therapy. Subsequently, the disease reactivated on several occasions, necessitating the intensification of vedolizumab treatment through an increase in the frequency of administration. However, the patient’s condition showed significant improvement over time. At the age of 9 years, the patient was in sustained clinical and laboratory remission using the combination of vedolizumab and methotrexate (Weighted Paediatric Disease Activity Index [5]: 17.5, fecal calprotectin: 4 µg/L). A follow-up endoscopy confirmed the achievement of endoscopic remission of the disease (Simple Endoscopic Score-Crohn’s disease [4]: 1). During the examination, four areas of predominantly low-grade tubulovillous adenomas (grade 3–4.1) were identified and removed. Detailed information on the treatment and course of the disease is shown in Supplementary Table 1.

Given these findings, the patient underwent comprehensive genetic testing with the informed consent of his parents. Primary analysis of whole-exome sequencing data in 2019 found no pathogenic variants in known inflammatory bowel disease-associated genes. In 2023, reanalysis using the megSAP pipeline and GSvar (IMGAG Tübingen) revealed a *de novo* indel variant c.1029 + 1_1029 + 5del in the *STXBP3* gene (NM_007269.4) in a heterozygous state [6]. The variant is classified as probably pathogenic according to ACMG criteria (PVS1_STR, PM2_SUP, PS2_MOD, PS3_SUP) [7]. Given the recurrent detection of dysplastic changes, genes associated with familial tumor predispositions were also examined. This did not identify another variant that could explain the patient’s condition. A similar finding of dysplastic changes in a patient with *STXBP3*-associated VEO-IBD was described in patient P2 in the study by Ouahed *et al*. [2].

## Discussion

In this case report, we present a novel patient diagnosed with *STXBP3*-associated hearing loss and VEO-IBD. During a re-analysis of whole-exome sequencing, we identified a novel *de novo* variant, c.1029 + 1_1029 + 5del. This variant has not been previously reported in the literature, nor is it listed in the population database GnomAD (version 4.1.0), or in the databases ClinVar and HGMD Professional. The SpliceAI predictor for this variant indicates a significant effect on splicing due to the disruption of the 5′ conserved splice site, likely leading to RNA degradation via nonsense-mediated decay. The variant is located at a previously described locus, where other variants c.1029 + 1G>A and c.1029delG, which also affect splicing according to SpliceAI, have been observed in patients with autosomal dominant inheritance of the disease. Reduction in protein expression for previously identified variants was confirmed through western blot analysis [2]. The progression of the disease in our patient aligns well with findings from previous studies, where all patients developed VEO-IBD. Additionally, sensorineural hearing loss was present in all patients with the autosomal dominant form of the disease. High-grade dysplasia in areas of chronic inflammation was also reported in one of these patients [2]. Unlike previously reported cases, this patient is currently responding well to a treatment regimen combining vedolizumab and methotrexate, without requiring corticosteroids. While other biologics, such as antitumor necrosis factor agents, may show improved pharmacokinetics when used with immunomodulators [8], in the case of vedolizumab, the drug’s gut-selective mechanism and lower rates of immunogenicity mean methotrexate is less likely to impact its pharmacokinetic profile [9]. However, methotrexate may offer additional immunosuppressive benefits, indirectly aiding clinical improvements rather than altering vedolizumab levels [10]. Therefore, the observed improvement in the patient’s condition is likely due to the combined effect, with vedolizumab playing a key role in enabling corticosteroid tapering [9].

## Supplementary Material

goaf053_Supplementary_Data
